# 

**Published:** 1896-04

**Authors:** 


### BOOKS RECEIVED.

Report of the Commissioner of Education for the year 1892-93. Volume II. Containing parts III. and IV. Washington : Government Printing Office. 1895.

Twenty-first Annual report of the State Board of Health of Michigan, for the year ending June 30, 1893. Henry B. Baker, M. D., Secretary. Lansing : Robert Smith & Co., State Printers. 1895.

Transactions of the Seventeenth Annual meeting of the American Laryngological Association, held in the City of Rochester, N. Y., June 17, 18 and 19, 1895. Edited by Charles H. Knight, M. D., Secretary. New York : D. Appleton & Co. 1896.

A Text-book upon the Pathogenic Bacteria for Students of Medicine and Physicians. By Joseph McFarland, M. D., Demonstrator of Pathological Histology and Lecturer on Bacteriology in the Medical Department of the University of Pennsylvania ; Pathologist to the Rush Hospital for Consumption and Allied Diseases. Octavo, pp. 359. With 113 illustrations. Price, $2.50 net. Philadelphia : W. B. Saunders, 925 Walnut street. 1896.

Infantile Mortality during Childbirth and its Prevention. By A. Brothers, B. S., M. D., Visiting Gynecologist to Beth Israel Hospital, New York ; Attending Gynecologist to the New York Clinic for Diseases of Women ; Instructor in Operative Gynecology at the New York Post-Graduate Medical School and Hospital, etc. Octavo, pp. viii.—179. Price, $1.50. Philadelphia : P. Blakiston, Son & Co., 1012 Walnut street. 1896.

Transactions of the American Microscopical Society. Eighteenth Annual meeting, held at Cornell University, Ithaca, N. Y., August 21, 22 and 23, 1895 Edited by William C. Krauss, M. D., Secretary, Buffalo, N. Y. Price, $2.50. The Wenborne-Sumner Co., Printers. 1896.

New Truths in Ophthalmology as developed by G. C. Savage, M. D., Professor of Ophthalmology in the Medical Department of the Vanderbilt University, etc. Pp. viii.—270. With fifty-eight illustrations. Third edition. Nashville, Tenn., 1896.

A Treatise on the Diseases of Infancy and Childhood. By J. Lewis Smith, M. D., Clinical Professor of Diseases of Children in the Bellevue Hospital Medical College, New York. New, eighth edition, thoroughly revised and rewritten and much enlarged. Handsome octavo of 983 pages, with 273 illustrations and four full-page plates. Cloth, $4.50; leather, $5.50. New York and Philadelphia: Lea Brothers & Co., Publishers. 1896.

The Toxic Amblyopias: their Symptoms, Pathology and Treatment. By George E. De Schweinitz, M. D., Clinical Professor of Ophthalmology, Jefferson Medical College, of Philadelphia. Octavo 240 pages, forty-one engravings and nine full-page colored plates. Limited edition. De luxe binding, $4.00, net. New York and Philadelphia: Lea Brothers & Co., Publishers. 1896.

Electricity in Electro-Therapeutics. By Edwin J. Houston, Ph. D., and A. E. Kennelly, Sc. D. Cloth, 412 pages, 128 illustrations. Price, $1.00. New York: The W. J. Johnston Co., Publishers, 253 Broadway.

